# Dual function of Gasdermin E: pyroptosis-mediated pan-cancer suppression versus HCC-specific oncogenic activity

**DOI:** 10.3389/fimmu.2025.1626311

**Published:** 2025-06-11

**Authors:** Gulijiamali Kahaer, Sirun Pan, Chengcheng Yang, Wenchen Xie, Yan Lu

**Affiliations:** Key Laboratory of Tropical Translational Medicine of Ministry of Education & Hainan Provincial Key Laboratory of Carcinogenesis and Intervention, School of Basic Medicine and Life Sciences, Hainan Medical University, Haikou, Hainan, China

**Keywords:** GSDME, dual function, pyroptosis, pan-cancer, HCC

## Abstract

Gasdermin E (GSDME), a key executor of pyroptosis, exerts a unique dual role in tumorigenesis, acting as both a tumor suppressor and a tumor-promoting factor. Due to promoter hypermethylation, GSDME is epigenetically silenced in most solid tumors, including gastric, colorectal, and breast cancers. Its activation triggers the release of inflammatory cytokines, such as IL-1β and IL-18, enhances CD8^+^ T cell infiltration, and improves chemosensitivity, thereby exerting potent tumor-suppressive effects. Hepatocellular carcinoma (HCC) displays an aberrant GSDME overexpression pattern, which promotes immune suppression and resistance to anti-PD-1 therapy through pyroptosis-independent mechanisms. Notably, specific interventions can activate GSDME-mediated pyroptosis in HCC, highlighting its functional plasticity in response to microenvironmental signaling networks. Current studies face three major challenges: elucidating the mechanisms underlying GSDME overexpression in HCC, clarifying the molecular hubs of pyroptosis-independent pro-tumor pathways, and developing precision strategies to control the functional switch of GSDME. Future studies should integrate single-cell multi-omics and spatial transcriptomics to establish a novel therapeutic paradigm based on “pyroptosis immunomodulation”, advancing cancer treatment from single-target inhibition toward multidimensional “microenvironment reprogramming”.

## 1 Introduction

**Figure 1:**
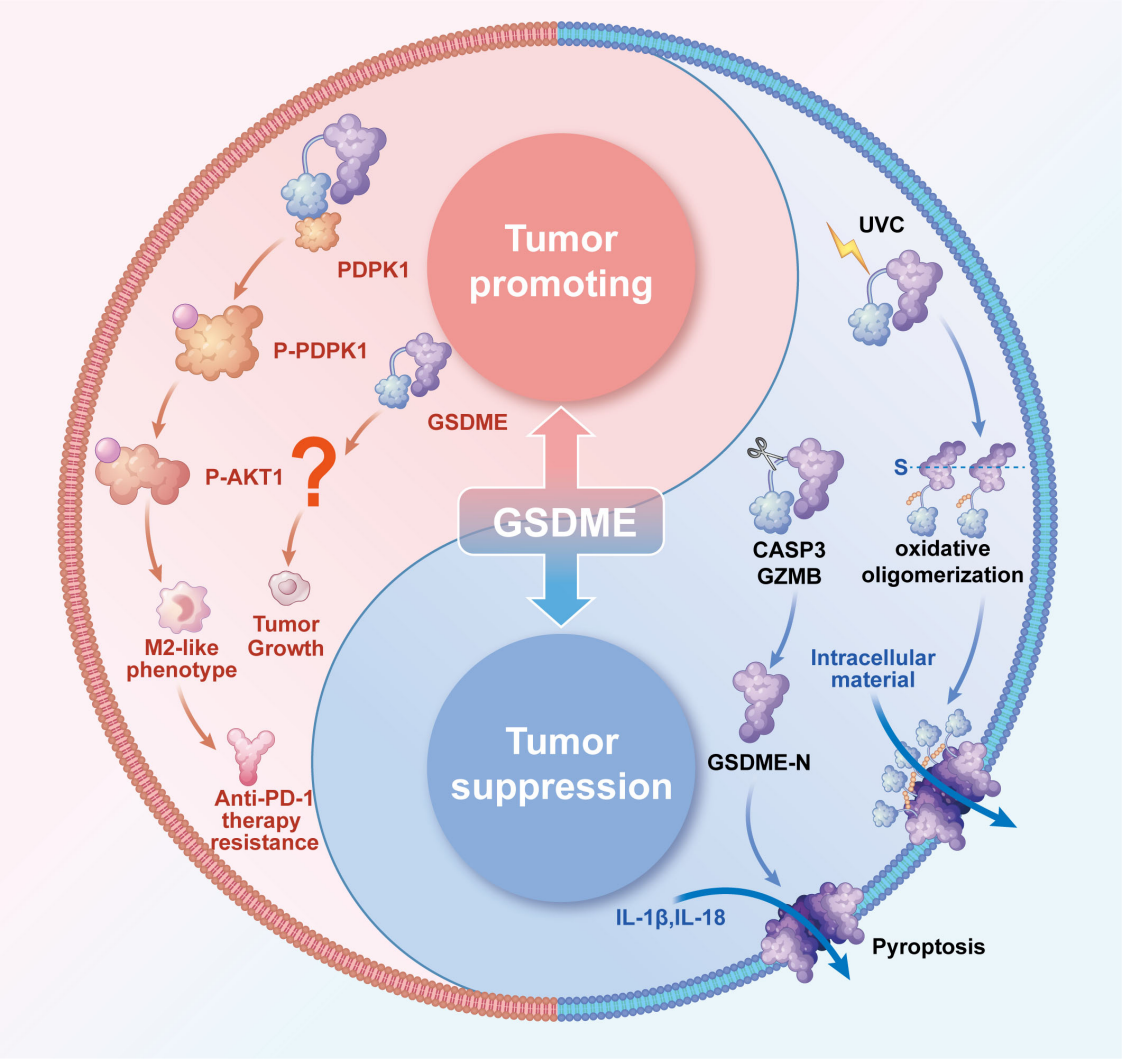
The dual blade function of GSDME in tumors. The Tumor-suppressive role of GSDME. In most solid tumors, including gastric, colorectal, cervical, and breast cancers, GSDME exerts tumor-suppressive effects through two distinct mechanisms of nanopore formation: Proteolytic Cleavage Pathway: Caspase-3 or GZMB specifically cleaves GSDME, releasing its N-terminal domain. This domain selectively binds membrane phospholipids and undergoes oligomerization to form transmembrane pores. Under UVC irradiation, full-length GSDME undergoes conformational changes, exposing its intrinsically pore-forming N-terminal domain. Subsequently, this domain forms pre-pore oligomers via disulfide bond-dependent oxidation, ultimately targeting and perforating the plasma membrane.Both pathways culminate in pyroptotic cell death, releasing inflammatory cytokines (e.g., IL-1β and IL-18) to activate antitumor immunity. The tumor-promoting role of GSDME. In HCC, GSDME is specifically overexpressed in TAMs, where it binds to PDPK1 to induce phosphorylation and subsequent activation of the PI3K-AKT pathway. This drives macrophage polarization toward an M2-like phenotype, which suppresses CD8^+^ T cell function and confers resistance to anti-PD-1 therapy. Emerging evidence suggests GSDME may also directly influence tumor growth, though the underlying mechanisms remain unclear. Collectively, GSDME exhibits dual context-dependent roles in cancer-inhibiting tumor progression in some contexts while promoting oncogenesis in others, with its functional outcome likely determined by tumor type and microenvironmental.

GSDME, an essential member of the Gasdermin family, plays a vital role in regulating pyroptosis, a type of programmed cell death associated with inflammatory responses. It does this by forming nanoscale membrane pores through two distinct pathways: one involves specific cleavage by Caspase-3 or granzyme B, while the other results from conformational changes induced by UVC irradiation, as shown in **Figure 1** (1–4). The cleaved GSDME-N-terminal domain possesses a unique ability to specifically recognize membrane phospholipids, leading to its oligomerization, which results in the formation of transmembrane pores that measure approximately 10–15 nm in diameter. Alternatively, when activated by UVC, the full-length GSDME exposes its N-terminal domain, which intrinsically possesses pore-forming activity, and undergoes a process of disulfide bond-dependent oxidative oligomerization to create prepores. These prepores subsequently target and perforate the plasma membrane, ultimately resulting in cell swelling and the induction of pyroptosis. Both of these activation pathways culminate in the release of inflammatory cytokines, such as IL-1β and IL-18, as depicted in **Figure 1** (2, 4).

Research has demonstrated that GSDME exists in two major isoform forms: the full-length GSDME (GSDME-FL) and the exon 8-splicing variant (GSDME-Δexon8). Currently identified GSDME genetic variants are all gain-of-function mutations, which induce exon 8 skipping, leading to premature termination of the open reading frame (ORF) and ultimately producing a truncated protein with significant cytotoxicity (5). Comparative studies by van Laer’s team demonstrated distinct subcellular localization patterns when wild-type GSDME and exon 8-deleted variant plasmids were transfected into COS-1 and HEK293T cells. The wild-type protein primarily localized in the cytoplasm, the variant protein exhibited dual cytoplasmic and plasma membrane distribution, accompanied by characteristic morphological changes including cell rounding and membrane blebbing (5). Further investigations revealed that the exon 8 skipping-generated truncated GSDME retains pyroptosis-inducing capability in mammalian cells, although with markedly reduced protein stability and shorter duration of biological effects compared to the classical GSDME-NT fragment (2). The precise molecular mechanisms by which these splicing variants regulate pyroptosis remain to be fully elucidated.

Notably, GSDME exhibits a fascinating and somewhat paradoxical ‘expression-function’ dichotomy across various malignancies: while it demonstrates epigenetic silencing and tumor-suppressive roles in the majority of solid tumors, including gastric, colorectal, and breast cancers, it paradoxically shows aberrant overexpression and drives malignant progression specifically in HCC (6). This tissue-specific paradox suggests that the biological functions of GSDME essentially reflect the dynamic interactions between the epigenetic landscapes and the microenvironmental signaling networks, with its ‘death signal hijacking’ mechanism providing novel perspectives for understanding the complexities of tumor heterogeneity and the varied responses observed in different cancer types (7).

## 2 Molecular mechanisms underlying GSDME-mediated tumor suppression

### 2.1 Epigenetic inactivation and suppression of tumorigenesis

The epigenetic inactivation of GSDME, which functions as a crucial tumor suppressor gene, is commonly observed in various types of cancers. Genome-wide methylation analyses have indicated that the hypermethylation of CpG islands located within the promoter region of GSDME serves as the primary mechanism responsible for its transcriptional silencing, a phenomenon that has been widely observed in multiple types of cancer, as documented in various studies (8–15). Furthermore, analyses of clinical samples have uncovered significantly elevated frequencies of GSDME methylation in cases of gastric cancer (52%, 46/89), colorectal cancer (65%, 65/100), and breast cancer, particularly when compared to their corresponding adjacent normal tissues (8–10). In addition, *in vitro* functional validation experiments have confirmed that treatment with the methyltransferase inhibitor 5-Aza-dC effectively reverses the suppression of GSDME expression in colorectal cancer cell lines, specifically in HCT116, HT29, and DLD-1 cell lines (11). Through the establishment of gene-edited cell models, researchers have discovered that the exogenous overexpression of GSDME significantly inhibits the proliferation of tumor cells and enhances their sensitivity to chemotherapeutic drugs, while conversely, the knockout of GSDME promotes tumor cell clonogenic formation and increases their invasive capabilities (11–13). Collectively, these lines of evidence elucidate the critical role of GSDME as a tumor suppressor gene, as well as the intricate epigenetic regulatory mechanisms that underlie its function.

### 2.2 Pyroptosis-mediated anti-tumor immunity

The intricate mechanism by which GSDME remodels the tumor immune microenvironment through the induction of pyroptosis has been substantiated by a multitude of comprehensive studies, with its core functions encompassing three pivotal aspects that are critical to understanding its role in cancer biology. First and foremost, the activation of GSDME significantly promotes the infiltration of immune cells into the tumor microenvironment through the release of inflammatory cytokines, such as IL-1β and IL-18. This release markedly enhances the tumor infiltration of crucial immune components, including CD8^+^ T cells and dendritic cells, thereby facilitating the transformation of immunologically “cold” tumors into “hot” microenvironments that are more conducive to immune attack (7). This phenomenon is particularly evidenced in osteosarcoma, where GSDME-mediated pyroptosis triggers a cascade of immune responses characterized by the release of proinflammatory cytokines, an increase in immune cell infiltration, and the activation of adaptive immune responses, ultimately leading to the establishment of a highly immunogenic tumor niche that can be targeted by the immune system. Secondly, GSDME has been shown to enhance the response to immunotherapy, as demonstrated by a pivotal study published in Nature in 2020, which revealed that GSDME-dependent cooperation between natural killer (NK) cells and CD8^+^ T cells significantly suppresses tumor progression in various models of melanoma and breast cancer (7). This underscores the potential of GSDME as a critical player in augmenting the efficacy of immunotherapeutic strategies. Lastly, GSDME improves chemosensitivity across a diverse range of cancer types. For instance, in triple-negative breast cancer, it plays a vital role in maintaining the cytotoxic function of CD8^+^ T cells, thereby enhancing the overall efficacy of treatment regimens (11, 12). Similarly, in non-small cell lung cancer, GSDME boosts the response to cisplatin by promoting the recruitment of T cells to the tumor site, which is essential for effective chemotherapy (16). These findings provide a robust theoretical foundation for the development of GSDME-modulated combination immunotherapy strategies, highlighting its potential as a transformative target in the fight against cancer.

Recent studies have also revealed the critical regulatory role of GSDME in tumor immunotherapy. Clinical evidence demonstrates a significant positive correlation between GSDME expression levels and immunotherapy responsiveness. A retrospective cohort study of non-small cell lung cancer (NSCLC) patients showed that patients with high GSDME expression in tumor tissues exhibited better treatment responses to PD-L1 inhibitor combined with chemotherapy, with significantly improved objective response rate (ORR) and a median progression-free survival (PFS) of 18.20 months, markedly longer than that of the low GSDME expression group (6.70 months) (HR = 0.37, 95% CI: 0.14-0.97; P = 0.0371) (17). Mechanistic studies indicate that GSDME-mediated pyroptosis can promote tumor antigen presentation and enhance T cell infiltration by releasing inflammatory factors such as IL-1β and IL-18, thereby improving the tumor immune microenvironment. These findings have been further validated in preclinical models of breast cancer and melanoma. GSDME deficiency leads to reduced infiltration of CD8^+^ T cells, NK cells, and tumor-associated macrophages in the tumor microenvironment, significantly impairing antitumor immune responses (7). These results suggest that GSDME not only participates in regulating the tumor immune microenvironment but may also serve as a potential biomarker for predicting the efficacy of immune checkpoint inhibitors.

## 3 The dual-edged sword function of GSDME in HCC

### 3.1 Tissue-specific aberrant expression

Unlike most cancers where GSDME is epigenetically silenced, HCC shows a strikingly different pattern with significant GSDME overexpression. This intriguing phenomenon is substantiated by comprehensive multi-omics analyses. For instance, transcriptomic data sourced from the TCGA and GEO databases clearly indicate that GSDME mRNA expression is significantly elevated in HCC when compared to normal liver tissue (**Table 1**) (18–23). Immunohistochemical analysis reveals a strikingly high positivity rate for GSDME in approximately 78% (289/371) of HCC specimens, far exceeding the 12% (6/50) positivity rate in adjacent non-tumor tissues (**Table 1**) (21, 22). These findings are reinforced by Western blot analyses showing consistent GSDME overexpression across multiple HCC cell lines, such as HepG2, HCCLM3, MHCC97H, LM3, SMMC-7721, BEL-7402, and Huh7 (**Table 1**) (21). Clinically, this overexpression has important implications research by De Schutter et al. links high GSDME levels with higher Edmondson tumor grades and poorer overall survival (OS) (HR=1.64, 95% CI:1.16-2.23; P=0.0051) (23). What makes this particularly intriguing is that GSDME appears to promote tumor growth in HCC, contrasting sharply with its tumor-suppressing role in other cancers. Indicating that HCC may have unique regulatory mechanisms.

**Table 1:** GSDME expression patterns and clinical significance in hepatocellular carcinoma.

Level	Findings	Clinical correlation	Reference
Gene	GSDME is upregulated in HCC (TCGA and GEO database)	GSDME is associated with poor overall survival (OS) (HR=1.64, 95% CI:1.16-2.23; P=0.0051)	(23)
	The expression of GSDME in tumor tissue is higher than that in normal tissue (TCGA and GEO database)	GSDME is associated with poor disease specific survival (DSS)	(21)
	GSDME is highly expressed in tumor tissue	GSDME is related to Edmondson grade	(6, 19)
Protein	Strong GSDME expression was observed in 78% (289/371) of HCC patients (THPA database; IHC)		(21)
	Overexpression of GSDME in seven HCC cell lines (HepG2, HuH7, HCCLM3, MHCC97H, LM3, SMMC-7721, BEL-7402)		(6, 21)

HCC, hepatocellular carcinoma; IHC, immunohistochemistry; THPA, the human protein atlas; OS, overall survival; HR, hazard ratio; DSS, disease specific survival.

The expression of GSDME is precisely regulated through multi-layered molecular mechanisms, and its functional heterogeneity across different tumor types may be associated with dynamic interactions between epigenetic modifications and transcription factors. Studies have demonstrated a significant negative correlation between hypermethylation of CpG islands in the GSDME promoter region and its mRNA expression levels in solid tumors such as gastric and colorectal cancers, establishing the critical role of DNA methylation in GSDME regulation (6). Epigenetic modifications also involve dynamic histone modifications, with researchers identifying significant enrichment of H3K4me3 histone marks at the GSDME promoter, suggesting chromatin remodeling may participates in its expression regulation. Research has found that ALKBH4 can transcriptionally suppress GSDME activation by inhibiting H3K4me3 histone modifications at its promoter region, thereby reducing tumor cell sensitivity to 5-FU treatment (9), findings that highlight the importance of histone modifications in GSDME regulation. At the transcription factor level, research has demonstrated that p53 can directly bind to conserved response elements situated 1–500 base pairs downstream of the transcription start site in the GSDME promoter, thereby activating its transcription (24). Concurrently, the transcription factor Sp1 enhances GSDME gene expression by specifically binding to the -36 to -28 region of its promoter (25). In HCC, the observed overexpression of GSDME may arise from the combined influence of epigenetic and transcriptional regulation. Specifically, promoter hypomethylation and H3K4me3 enrichment create a permissive environment for transcription, while mutant p53 and other transcription factors further amplify expression levels, particularly under inflammatory or hypoxic microenvironments. Moreover, chronic inflammation-driven STAT1 signaling and metabolic disturbances may indirectly elevate GSDME expression by modifying epigenetic patterns. However, the precise regulatory mechanisms underlying these phenomenons remain to be fully elucidated and warrant further investigation through comprehensive methylation profiling and transcription factor binding assays.

### 3.2 Pyroptosis-independent oncogenic mechanisms in HCC

While the precise mechanisms underlying GSDME’s tumor-promoting function in HCC remain incompletely characterized, accumulating evidence implicates two key pathways as central to this oncogenic process. Firstly, there is the remodeling of the immune microenvironment; single-cell sequencing studies have revealed a specific overexpression of GSDME in tumor-associated macrophages (TAMs) (26). This overexpression drives the polarization of these macrophages towards the M2 phenotype through the activation of the PI3K-AKT signaling pathway, which, in turn, leads to the suppression of CD8^+^ T cell function, as illustrated in **Figure 1** (26). Secondly, GSDME directly promotes tumorigenesis; *in vivo* studies confirm that GSDME knockout markedly decreases tumor volume across multiple HCC models. Nevertheless, the exact molecular mechanism underlying the tumor growth suppression effect following GSDME knockout remains to be fully elucidated (27, 28). Notably, GSDME exerts its oncogenic role in HCC through pyroptosis-independent mechanisms, representing a paradigm shift in our understanding of gasdermin family proteins in cancer biology. While GSDME is traditionally recognized for its pyroptosis-inducing capacity in response to chemotherapeutic agents, in HCC, its tumor-promoting effects manifest through alternative pathways that bypass characteristic pyroptotic cell death markers such as cell swelling, pore formation, and massive IL-1β release. This non-canonical activity likely explains both GSDME’s upregulated expression during HCC progression and the diminished anti-PD-1 therapy response in patients with high GSDME levels (26). As for its specific tumor-promoting mechanisms in hepatocellular carcinoma, substantial experimental evidence remains to be established.

### 3.3 Pyroptosis-dependent tumor suppression pathways in HCC

The emerging evidence confirms that GSDME still maintains its tumor suppressor function in HCC under specific therapeutic conditions. A prime example is oxaliplatin, a platinum-based chemotherapeutic agent that actively induces pyroptosis through caspase-3/GSDME axis activation. Importantly, this mechanism exerts anti-tumor effects through dual pathways, directly inducing programmed cell death in tumor cells and promoting CD8^+^ T cell infiltration into the tumor microenvironment by activating the p38/MAPK signaling pathway (29). The two synergistically enhance anti-tumor effects. The histone deacetylase inhibitor CXD101 exerts its therapeutic effect through another mechanism. It promotes GSDME dependent pyroptosis by activating STAT1 signaling and significantly enhances the sensitivity of HCC cells to immunotherapy (30). Meanwhile, studies have found that SIRT1 silencing can release inhibition of GSDME-N, leading to typical cell pyroptosis phenomena including significant membrane foaming and significant lactate dehydrogenase (LDH) release (31). Collectively, these findings demonstrate that modulation of key signaling pathways can restore GSDME-dependent tumor suppression in HCC. This establishes a mechanistic rationale for developing GSDME-targeted therapeutic strategies in liver cancer.

## 4 Summary and outlook

GSDME demonstrates a context-dependent dual role in tumor biology, exhibiting both tumor-suppressive and oncogenic activities that highlight the functional plasticity of this molecule within complex tumor microenvironment regulatory networks. In most solid tumors, GSDME exerts tumor-suppressive effects. It mediates pyroptosis, enhances the release of pro-inflammatory factors, remodels the immune microenvironment, and enhances chemosensitivity (32–34). In contrast, HCC demonstrates an inverse regulatory pattern. Aberrant GSDME overexpression in HCC promotes immune suppression and resistance to anti-PD-1 therapy through pyroptosis-independent mechanisms (26). Interestingly, if drugs such as oxaliplatin are used to activate the caspase-3/GSDME pathway, the pyroptosis process can be restarted, promoting T cells to enter the tumor area and exert their effects (23). So, if organ specific targeted regulation or programmable spatiotemporal specific activation/inhibition of GSDME can be achieved, it will provide a new breakthrough for tumor treatment.

In recent years, some progress has been made in the development of innovative GSDME targeted delivery systems and small molecule modulators. A research team led by Gui Jun at Renji Hospital, Shanghai Jiao Tong University School of Medicine demonstrated that intratumoral delivery of lipid nanoparticle-encapsulated *Gsdme* mRNA significantly suppressed tumor progression in murine models of melanoma and colorectal carcinoma. Mechanistic studies revealed that Gsdme mRNA-triggered pyroptosis released creatine as a novel metabolite-derived damage-associated molecular pattern (DAMP), which activated the type I interferon signaling pathway in monocytes to potentiate CD8^+^ T cell-mediated antitumor immunity (35). The research group led by Shuai Xintao at Sun Yat-sen University developed GM@LR nanoliposomes that achieved co-delivery of gsdme encoding genetic material and manganese carbonyl (MnCO). This combinatorial approach demonstrated marked therapeutic efficacy in triple-negative breast cancer models through coordinated activation of pyroptotic cell death and STING pathway signaling (36). Notable progress has also been made in small-molecule modulator development. Researchers led by Li Ping and Chen Jun at China Pharmaceutical University identified ponatinib and perifosine as GSDME-N-terminal agonists through high-throughput screening. These compounds not only directly triggered tumor cell pyroptosis but also exhibited remarkable synergistic antitumor effects when combined with PD-1 blockade inhibitors (37). On the other hand, methylcobalamin exhibits distinct inhibitory effects on GSDME activation. The conformational rearrangement of Methylcobalamin exposes its central cobalt atom, which subsequently coordinates with Cys180 in the GSDME N-terminal domain. This cobalt-thiolate interaction sterically hinders caspase-3-mediated cleavage at Asp270, effectively suppressing pyroptotic pore (38). The research team at Xiamen University serendipitously discovered that mannose suppresses pyroptosis through a previously unrecognized metabolic-immune crosstalk. Mannose elevates intracellular GlcNAc-6P levels, which allosterically activates AMPK. AMPK mediated phosphorylation of GSDME at Ser103 sterically impedes caspase-3 binding at the cleavage site, effectively blocking GSDME processing and subsequent pore formation (39). Although these strategies have performed well in preclinical models such as colorectal cancer and breast cancer, bottlenecks such as insufficient delivery efficiency of nanoparticles and poor targeting of small molecule drug tissues still need to be solved. Future research should focus on developing smart delivery systems and exploring combination therapies integrating GSDME modulators with immunotherapy and epigenetic drugs to accelerate clinical translation. These advancements are expected to overcome current limitations and provide new therapeutic opportunities.

Current research on GSDME function in HCC grapples with several critical unresolved issues. A primary challenge lies in delineating the precise mechanisms underlying aberrant GSDME activation in HCC cells, particularly the role of epigenetic modifications that are fundamental to understanding its promotion of HCC. Equally pressing is the identification of key molecular mediators facilitating GSDME mediated, pyroptosis independent oncogenesis, with special emphasis on tumor cell communication with immune populations like macrophages through extracellular vesicle trafficking or cytokine signaling interactions. Furthermore, developing technologies capable of precisely modulating the functional duality of GSDME to balance its tumor promoting versus tumor suppressing activities remains an essential objective in the field.

Addressing these challenges will likely require integration of some cutting edge methodologies. Spatial multi-omics platforms, for instance, can simultaneously analyze gene expression patterns and protein localization within the tumor microenvironment, enabling systematic mapping of dynamic interrelationships between GSDME and immune cell infiltration. Emerging spatial profiling technologies like CITE-seq (high-plex protein and whole transcriptome co-mapping), despite current limitations in multiplex protein detection capacity, hold significant promise for elucidating GSDME-immune interactions in HCC. As this methodology matures, its capacity to simultaneously resolve both proteomic and transcriptomic landscapes at single-cell resolution within intact tumor microenvironments will enable precise mapping of spatial correlations between GSDME expression and immune infiltration (40). The multimodal three omics spatial mapping technique can integrate epigenetic, transcriptomic, and proteomic data to systematically analyze the functional transition status of GSDME in different tumor regions. For example, tracking the molecular driving factors behind the dependent switching between pro tumor and tumor suppressive phenotypes of GSDME in different tumor regions (41). To investigate GSDME’s upstream/downstream regulatory mechanisms, Perturb-DBiT based spatially resolved panoramic *in vivo* CRISPR screening allows high throughput genetic perturbation coupled with spatial transcriptomic analysis in living models, facilitating genome wide identification of key targets that cooperatively regulate pyroptosis or immune evasion with GSDME (42, 43). These technologies will elucidate the tissue-specific functional heterogeneity of GSDME while providing a methodological framework for developing microenvironment-responsive precision therapies. Importantly, by deciphering these regulatory networks, this work may enable the design of innovative HCC treatment strategies based on the “pyroptosis immunoediting” paradigm. Breakthroughs in this field not only surpass the traditional research framework of “functional loss” of tumor suppressor genes, but also have the potential to drive a paradigm shift in cancer treatment from a single targeted model to “multidimensional microenvironment reprogramming”.

## References

[1] Shi J Gao W Shao F. Pyroptosis: gasdermin-mediated programmed necrotic cell death. Trends Biochem Sci. (2016) 42:245–54. doi: 10.1016/j.tibs.2016.10.004 27932073

[2] Wang Y Gao W Shao F Ding J Liu W He H. Chemotherapy drugs induce pyroptosis through caspase-3 cleavage of a gasdermin. Nature. (2017) 547:99–103. doi: 10.1038/nature22393 28459430

[3] Zhou B Jiang ZH Dai MR Ai YL Xiao L Zhong CQ. Full-length GSDME mediates pyroptosis independent from cleavage. Nat Cell Biol. (2024) 26:1545–57. doi: 10.1038/s41556-024-01463-2 38997456

[4] Zhang E Healy L Du G Wu H. Cleavage-independent GSDME activation by UVC. Nat Cell Biol. (2024) 26:1377–9. doi: 10.1038/s41556-024-01470-3 PMC12587310 39085377

[5] Van Laer L Vrijens K Thys S Van Tendeloo VF Smith RJ Van Bockstaele DR. DFNA5: hearing impairment exon instead of hearing impairment gene? J Med Genet. (2004) 41:401–6. doi: 10.1136/jmg.2003.015073 PMC1735793 15173223

[6] Lu Y Xu J Lin H Zhu M Li M. Gasdermin E mediates pyroptosis in the progression of hepatocellular carcinoma: a double-edged sword. Gastroenterol Rep(Oxf). (2024) 12:goae102. doi: 10.1093/gastro/goae102 39526199 PMC11549059

[7] Zhang Z Zhang Y Xia S Kong Q Li S Liu X. Gasdermin E suppresses tumour growth by activating anti-tumour immunity. Nature. (2020) 579:415–20. doi: 10.1038/s41586-020-2071-9 PMC7123794 32188940

[8] Akino K Toyota M Suzuki H Imai T Maruyama R Kusano M. Identification of DFNA5 as a target of epigenetic inactivation in gastric cancer. Cancer Sci. (2007) 98:88–95. doi: 10.1111/j.1349-7006.2006.00351.x 17083569 PMC11158324

[9] Jiang X Zhu Z Ding L Du W Pei D. ALKBH4 impedes 5-FU Sensitivity through suppressing GSDME induced pyroptosis in gastric cancer. Cell Death Dis. (2024) 15:435. doi: 10.1038/s41419-024-06832-1 38902235 PMC11189908

[10] Luo B Zhang S Yu X Tan D Wang Y Wang M. Gasdermin E benefits CD8^+^T cell mediated anti-immunity through mitochondrial damage to activate cGAS-STING-interferon β axis in colorectal cancer. biomark Res. (2024) 12:59. doi: 10.1186/s40364-024-00606-9 38853246 PMC11163757

[11] Kim MS Chang X Yamashita K Nagpal JK Baek JH Wu G. Aberrant promoter methylation and tumor suppressive activity of the DFNA5 gene in colorectal carcinoma. Oncogene. (2008) 27:3624–34. doi: 10.1038/sj.onc.1211021 18223688

[12] Lin J Lyu Z Feng H Xie H Peng J Zhang W. CircPDIA3/miR-449a/XBP1 feedback loop curbs pyroptosis by inhibiting palmitoylation of the GSDME-C domain to induce chemoresistance of colorectal cancer. Drug Resist Update. (2024) 76:101097. doi: 10.1016/j.drup.2024.101097 38861804

[13] Fujikane T Nishikawa N Toyota M Suzuki H Nojima M Maruyama R. Genomic screening for genes upregulated by demethylation revealed novel targets of epigenetic silencing in breast cancer. Breast Cancer Res Treat. (2010) 122:699–710. doi: 10.1007/s10549-009-0600-1 19859801

[14] Wang H Rong X Zhao G Zhou Y Xiao Y Ma D. The microbial metabolite trimethylamine N-oxide promotes antitumor immunity in triple-negative breast cancer. Cell Metab. (2022) 34:581–94. doi: 10.1016/j.cmet.2022.02.010 35278352

[15] Fu C Ji W Cui Q Chen A Weng H Lu N. GSDME-mediated pyroptosis promotes anti-tumor immunity of neoadjuvant chemotherapy in breast cancer. Cancer Immunol Immunother. (2024) 73:177. doi: 10.1007/s00262-024-03752-z 38954046 PMC11219631

[16] Peng Z Wang P Song W Yao Q Li Y Liu L. GSDME enhances Cisplatin sensitivity to regress non-small cell lung carcinoma by mediating pyroptosis to trigger antitumor immunocyte infiltration. Signal Transduct Target Ther. (2020) 5:159. doi: 10.1038/s41392-020-00274-9 32839451 PMC7445264

[17] Xuzhang W Lu T Jin W Yu Y Li Z Shen L. Cisplatin-induced pyroptosis enhances the efficacy of PD-L1 inhibitor in small-cell lung cancer via GSDME/IL12/CD4Tem axis. Int J Biol Sci. (2024) 20:537–53. doi: 10.7150/ijbs.89080 PMC10758111 38169676

[18] Li G Zhang D Liang C Liang C Wu J. Construction and validation of a prognostic model of pyroptosis related genes in hepatocellular carcinoma. Front Oncol. (2022) 12:1021775. doi: 10.3389/fonc.2022.1021775 36338707 PMC9633965

[19] Gao X Wang WX Zhang XL. A novel pyroptosis risk model composed of NLRP6 effectively predicts the prognosis of hepatocellular carcinoma patients. Cancer Med. (2023) 12:808–23. doi: 10.1002/cam4.4898 PMC9844607 35651286

[20] De Schutter E Croes L Ibrahim J Pauwels P Op de Beeck K Vandenabeele P. GSDME and its role in cancer: From behind the scenes to the front of the stage. Int J Cancer. (2021) 148:2872–83. doi: 10.1002/ijc.33390 33186472

[21] Hu K Xu Z Yao L Yan Y Zhou L Li J. Integrated analysis of expression, prognostic value and immune infiltration of GSDMs in hepatocellular carcinoma. Aging (Albany NY). (2021) 13:24117–35. doi: 10.18632/aging.203669 PMC8610125 34731088

[22] Shen Q Jiang Y Hu X Du Z. A newly identified pyroptosis-related gene signature for predicting prognosis of patients with hepatocellular cancer. Transl Cancer Res. (2022) 11:3175–86. doi: 10.21037/tcr-22-366 PMC9552084 36237236

[23] Zhang X Zhang P An L Sun N Peng L Tang W. Miltirone induces cell death in hepatocellular carcinoma cell through GSDME-dependent pyroptosis. Acta Pharm Sin B. (2020) 10:1397–413. doi: 10.1016/j.apsb.2020.06.015 PMC7488361 32963939

[24] Chen W Yang KB Zhang YZ Lin ZS Chen JW Qi SF. Synthetic lethality of combined ULK1 defection and p53 restoration induce pyroptosis by directly upregulating GSDME transcription and cleavage activation through ROS/NLRP3 signaling. J Exp Clin Cancer Res. (2024) 43:248. doi: 10.1186/s13046-024-03168-8 39215364 PMC11363528

[25] Pan J Li Y Gao W Jiang Q Geng L Ding J. Transcription factor Sp1 transcriptionally enhances GSDME expression for pyroptosis. Cell Death Dis. (2024) 15:66. doi: 10.1038/s41419-024-06455-6 38238307 PMC10796635

[26] Chen S Zhang P Zhu G Wang B Cai J Song L. Targeting GSDME-mediated macrophage polarization for enhanced antitumor immunity in hepatocellular carcinoma. Cell Mol Immunol. (2024) 21:1505–21. doi: 10.1038/s41423-024-01231-0 PMC11607431 39496854

[27] Liang X Liu Q Zhu S Li Z Chen H Su Z. GSDME has prognostic and immunotherapeutic significance in residual hepatocellular carcinoma after insufficient radiofrequency ablation. Transl Oncol. (2024) 39:101796. doi: 10.1016/j.tranon.2023.101796 37862939 PMC10589398

[28] Chen X Yang M Wang L Wang Y Tu J Zhou X. Identification and *in vitro* and *in vivo* validation of the key role of GSDME in pyroptosis-related genes signature in hepatocellular carcinoma. BMC Cancer. (2023) 23:411. doi: 10.1186/s12885-023-10850-1 37149620 PMC10164321

[29] Deng M Zhao R Zou H Guan R Wang J Lee C. Oxaliplatin induces pyroptosis in hepatoma cells and enhances antitumor immunity against hepatocellular carcinoma. Br J Cancer. (2025) 132:371–83. doi: 10.1038/s41416-024-02908-z PMC11832738 39748129

[30] Tu Y Wu H Zhong C Liu Y Xiong Z Chen S. Pharmacological activation of STAT1-GSDME pyroptotic circuitry reinforces epigenetic immunotherapy for hepatocellular carcinoma. Gut. (2025) 74:613–27. doi: 10.1136/gutjnl-2024-332281 PMC12013592 39486886

[31] Liu D Liu J Liu K Hu Y Feng J Bu Y. SIRT1 inhibition-induced mitochondrial damage promotes GSDME-dependent pyroptosis in hepatocellular carcinoma cells. Mol Biotechnol. (2024) 66:3628–39. doi: 10.1007/s12033-023-00964-z PMC11564359 38044396

[32] Hu Y Liu Y Zong L Zhang W Liu R Xing Q. The multifaceted roles of GSDME-mediated pyroptosis in cancer: therapeutic strategies and persisting obstacles. Cell Death Dis. (2023) 14:836. doi: 10.1038/s41419-023-06382-y 38104141 PMC10725489

[33] Fu J Li D Zhang L Maghsoudloo M Cheng J Fu J. Comprehensive analysis, diagnosis, prognosis, and cordycepin (CD) regulations for GSDME expressions in pan-cancers. Cancer Cell Int. (2024) 24:279. doi: 10.1186/s12935-024-03467-2 39118110 PMC11312966

[34] Zhang D Chen Y Sun Y Xu H Wei R Zhou Y. Gambogic acid induces GSDME dependent pyroptotic signaling pathway via ROS/P53/Mitochondria/Caspase-3 in ovarian cancer cells. Biochem Pharmacol. (2025) 232:116695. doi: 10.1016/j.bcp.2024.116695 39643123

[35] Li H Lu J Tan S Jiang T He X Qiao W. *In situ* delivery of Gasdermin E mRNA promotes antitumor immunity via creatine-elicited type interferon signaling in monocytes. Cancer Immunol Res. (2025). 13(6):939–56. doi: 10.1158/2326-6066.CIR-24-0834 40168141

[36] Zhong H Chen G Li T Huang J Lin M Li B. Nanodrug Augmenting Antitumor Immunity for Enhanced TNBC Therapy via Pyroptosis and cGAS-STING Activation. Nano Lett. (2023) 23:5083–91. doi: 10.1021/acs.nanolett.3c01008 37220198

[37] Liu Y Zhang X Zhang P He T Zhang W Ma D. A high-throughput luciferase reporter assay for screening potential gasdermin E activators against pancreatic cancer. Acta Pharm Sin B. (2023) 13:4253–72. doi: 10.1016/j.apsb.2023.07.018 PMC10548051 37799380

[38] Xu W Wang Y Cui S Zheng Q Lin Y Cui Q. Methylcobalamin protects against liver failure via engaging gasdermin E. Nat Commun. (2025) 16:1233–49. doi: 10.1038/s41467-024-54826-6 PMC11785938 39890804

[39] Ai YL Wang WJ Liu FJ Fang W Chen HZ Wu LZ. Mannose antagonizes GSDME-mediated pyroptosis through AMPK activated by metabolite GlcNAc-6P. Cell Res. (2023) 12):904–22. doi: 10.1038/s41422-023-00848-6 PMC10709431 37460805

[40] Liu Y DiStasio M Su G Asashima H Enninful A Qin X. High-plex protein and whole transcriptome co-mapping at cellular resolution with spatial CITE-seq. Nat Biotechnol. (2023) 41:1405–9. doi: 10.1038/s41587-023-01676-0 PMC10567548 36823353

[41] Fan R Zhang D Rodríguez-Kirby L Lin Y Song M Wang L. Spatial dynamics of mammalian brain development and neuroinflammation by multimodal tri-omics mapping. Res Sq. (2024) 12:rs.3.rs–4814866. doi: 10.21203/rs.3.rs-4814866/v1. Preprint.

[42] Baysoy A Tian X Zhang F Renauer P Bai Z Shi H. Spatially Resolved *in vivo* CRISPR Screen Sequencing via Perturb-DBiT. bioRxiv. (2024) 19:2024.11.18.624106. doi: 10.1101/2024.11.18.624106. Preprint.

[43] Enninful A Zhang Z Klymyshyn D Zong H Bai Z Farzad N. Integration of imaging-based and sequencing-based spatial omics mapping on the same tissue section via DBiTplus. Res Sq. (2024) 11:rs.3.rs–5398491. doi: 10.21203/rs.3.rs-5398491/v1 PMC13541625 41540123

